# Novel antimicrobial coating for hernia meshes

**DOI:** 10.3389/fcimb.2024.1383680

**Published:** 2024-10-22

**Authors:** Klaus Dieter Kühn, Débora C. Coraça-Huber, Michael Erdtmann, Gerwin A. Bernhardt, Christian Fölsch

**Affiliations:** ^1^ Department of Orthopaedics and Trauma, Medical University Graz, Graz, Austria; ^2^ Experimental Orthopaedics, Medical University Innsbruck, Innsbruck, Austria; ^3^ QC Department, Hemoteq AG, Würselen, Germany; ^4^ Division of General Surgery, Department of Surgery, University of Graz, Graz, Austria; ^5^ Department of Orthopaedic Surgery, Universitätsklinikum Gießen und Marburg (UKGM), Justus-Liebig-University, Gießen, Germany

**Keywords:** hernia meshes, gentamicin palmitate, chlorhexidine palmitic acid, chlorhexidine palmitate, infection, biofilm

## Abstract

**Purpose:**

Antibiotic coating for several medical devices has been carried out; however, there are only few studies about coating hernia meshes with antimicrobial substances. In this study we checked the capacity of different commercially available hernia meshes to act as drug carrier.

**Methods:**

The meshes were coated with gentamicin palmitate, chlorhexidine palmitic acid and chlorhexidine palmitate. The coating mass and subsequent *in vitro* delivery rate were evaluated for gentamicin palmitate by fluorescence polarization. For Chlorhexidine coated devices the coating mass was determined by weighing. The *in vitro* delivery rate was determined by UV absorption (255 nm). The interaction of each mesh to the different coating substances was observed by scanning electron microscopy.

**Results:**

1. Certain uniformity was observed on the quantity of chlorhexidine coating the surface of each mesh used when compared with gentamicin palmitate coating. 2.We did not detect significant difference between the amounts of gentamicin palmitate released from each mesh. 3. The release of chlorhexidine palmitate and chlorhexidine palmitic acid from UltraPro™ and Mersilene™ were significantly higher (p<0.05) in comparison with the other two meshes. 4. The coating substances covered the surface of the fibers without damaging its structure. 5. The coating substances were distributed all along the fibers in all samples.

**Conclusions:**

We suggest the use of chlorhexidine palmitate and chlorhexidine palmitic acid, as well as gentamicin palmitate, for coating of hernia meshes aiming prevention of infections. Further investigation of the bactericidal effect of coated hernia meshes against biofilm form of *S. aureus* and other device-related infections is suggested.

## Introduction

Hernia repair is one of the most frequently performed surgical procedures worldwide. Current hernia guidelines recommend the use of meshes for hernia repair whenever possible (Sanders et al., 2023). Moreover, the ongoing trend towards minimally invasive surgery leads to an increase of laparoscopic procedures which require the use of meshes. The number of hernias, especially incisional hernias, tends to increase because of the demographic evolution with more older patients needing abdominal surgical procedures resulting in a higher number of incisional hernias after laparotomy. On the other hand, obesity and overweight as a cause for higher intraabdominal pressure is increasing too in the last decades leading to higher hernia incidence. Additionally, recurrences after abdominal hernia repairs with conventional suturing demand for re-repairs using meshes. Beside the use in hernia surgery meshes are used for abdominal reinforcement after abdominal wall defects due to prior trauma or oncologic surgical interventions (Majumder et al., 2015). According to current guidelines meshes can also be used in contaminated or clean contaminated wounds however with a higher rate of of mesh infections (Blatnik et al., 2017). Many different hernia meshes are on the market composed of several biomaterials (Mirel et al., 2022). Partially absorbable polyglactin/polypropylene, polytetrafluoroethylene/polypropylene, as well as pure polypropylene meshes have been successfully used for hernia repair (Stremitzer et al., 2010). To avoid hernia recurrence current guidelines, recommend the use of macroporous (light weight), monofilament meshes. Most infections especially superficial wound infections can be treated with antibiotic therapy. However, in immune compromised and critically ill patients, mesh-related infections are a challenge for patients and surgeons necessitating revisional surgery with mesh removal and long hospitalizations (Majumder et al., 2016, Mirel et al., 2022).

Meshes which are colonized by bacteria lead to implant-related infections. The adhering bacteria in these cases can evade host defenses by forming biofilms. Bacteria in biofilms are encased in a polysaccharide matrix, which provides them with protection against the hosts defenses, antimicrobial drugs and biocides (Cangui-Panchi et al., 2022; Cangui-Panchi et al., 2023). Some bacteria express specific surface-associated proteins that allow the organisms to interact specifically with inflammatory proteins of the host cell, such as fibronectin, fibrinogen and collagen (Brady et al., 2008; Luong et al., 2009; Coraca-Huber et al., 2012). *Staphylococcus epidermidis* and *Staphylococcus aureus* are the bacteria which mostly colonize implant surfaces (Christensen et al., 1989; Coraca-Huber et al., 2013a). Removal of the infected mesh is recommended if the infection could not be resolved by antibiotic therapy (Szczerba and Dumanian, 2003; Fawole et al., 2006; Jezupovs and Mihelsons, 2006). In addition, mesh removal can result in hernia recurrence necessitating subsequent surgical procedures (Szczerba and Dumanian, 2003; de Vries Reilingh et al., 2004; Langer et al., 2005).

Antibiotics delivered from an implanted biomaterial may be potentially used to prevent infections caused by biofilm formation, providing high concentrations of antibiotics at the surgical site without local or systemic toxicity (Coraca-Huber et al., 2013b). Gentamicin sulfate (GS) salt is commonly used antibiotic for local application in surgery. Gentamicin base (GB) consists of a mixture of gentamicin C1, C1a and C2 a + b. Gentamicin sulfate is highly water soluble (Vogt et al., 2005). This substance can be used as a coating material for biomaterials and tissues by turning the water-soluble GS into a low-soluble gentamicin fatty acid salt (converting gentamicin sulfate to gentamicin palmitate; GP) (Kühn et al., 2003; Kuhn et al., 2008; Coraca-Huber et al., 2013b). Also, the same technique can be applied to antiseptic substances as chlorhexidine converting it to chlorhexidine palmitate or chlorhexidine palmitic acid (Vogt et al., 2005).

Antibiotic coating for intravenous catheters and some orthopedic devices has been carried out and are successful; however, there are only few studies about coating hernia meshes with antimicrobial substances.

The aim of this study was to evaluate the capacity of different commercially available hernia meshes to act as drug carrier for antimicrobial agents.

## Materials and methods

The meshes were coated with gentamicin palmitate, chlorhexidine palmitic acid and chlorhexidine palmitate. The coating mass and subsequent *in vitro* delivery rate were evaluated for each substance. The interaction of each mesh to the different coating substances was observed by scanning electron microscopy.

### Antibiotic and antiseptic solutions

Solutions of gentamicin palmitate (GP) (1g/mL, 4% in MeOH), chlorhexidine palmitate (Chl-P) 40,2g/mL, 4,7% in methanol) and chlorhexidine palmitic acid (Chl-PA) (37mg/mL, 4,5% in methanol) were prepared.

### Hernia mesh samples

For this study, hernia meshes were cut into 1x1 cm pieces.

### Pilot test for validation of coating method

Prior to the coating of different hernia meshes with all three solutions, a pilot test was carried out for validation of the methods used. Coating study with GP and Optilene meshes was carried out to determine the dependency of immersion time on coating deposition. Coating times between 30 and 600s were investigated. The coating amount was directly dependent on immersion time.

Bone cement containing gentamicin sulfate (PMMA, Palacos R+G, Heraeus Medical GmbH, Wehrheim, Germany) and samples of PerioChip^®^ (contains 2.5 mg of chlorhexidine gluconate in a biodegradable matrix of hydrolyzed gelatin; Dexcel Pharma GmbH; Alzenau; Germany) were used as reference. Three tests were carried out: (1) coating capacity; (2) gentamicin delivery rate in comparison to PMMA bone cement and (3) chlorhexidine delivery rate in comparison to PerioChip^®^.

### Hernia meshes

Then, UltraPro™ mesh composed of 75% polypropylene and 25% poliglecaprone (Johnson & Johnson Medical GmbH, Norderstedt, Germany); Mersilene™ mesh composed of polyester mesh (Johnson & Johnson Medical GmbH, Norderstedt, Germany); DynaMesh- IPOM^®^ composed of polyvinylidene fluoride and polypropylene (FEG Textiltechnik Forschungs- und Entwicklungsgesellschaft GmbH, Aachen, Germany); Parietene ™ mesh composed of polypropylene (Covidien plc, Duplin, Ireland); and C- QUR ™ mesh composed of polypropylene with bio-absorbable omega-3 (Atrium Europe B.V., Mijdrecht, The Netherlands) were used.

### Coating of the hernia meshes

All meshes used were coated by immersion in methanolic solution containing the substances.

The meshes were removed from the original packaging, cut in pieces of 1 cm^2^, weighed and immersed in the GP, Chl-P and Chl-PA solutions for coating.

For each combination of hernia material and coating solution a pilot study was carried out with regard to immersion time. Intervals of 30, 60, 120 and 200s were investigated. After this time period samples were removed from the solution and dried overnight at room temperature (RT). Then the mesh pieces were weighed again.

For subsequent elution studies, samples with Chl-P and CHl-PA were immersed for 200 s.

All coatings are carried out at Hemoteq AG, Würselen, Germany.

### Antibiotic and antiseptic elution rate

For gentamicin release each coated sample was added to 2 ml phosphate buffer (PBS) pH 7.4 and incubated at 37°C for 1 to 7 days. At each interval the samples were removed from PBS, dried at RT and transferred to fresh PBS. To determine the amount released, at each interval 100μl of each elution were analysed for fluorescence polarization using a TDx analyser (Abbott TDx system, Abbott Park, IL, USA). The elution rate was measured in µg/specimen. For GP coated Optilene^®^ antibiotic loaded PMMA cement was used as reference.

For chlorhexidine release each coated sample was added to 2 ml phosphate buffer (PBS) pH 7.4 and incubated at 37°C.At each interval the samples were removed from PBS, dried at RT and transferred to fresh PBS. Time points were 2, 4, 24. 48, 72, 144, 168 192 240 and 312 h after incubation start. To determine the chlorhexidine amount released, the PBS supernatant was added to a 1cm quartz cell, and the absorption analysed at 255 nm. It was converted in µg released substance/specimen ³ using a linear calibration function.

For Optilene^®^ coated with chlorhexidine we used Periochip^®^ as reference.

### Scanning electron microscopy

Images were obtained from coated and uncoated samples (as control) by using a tabletop scanning electron microscope (SEM) Hitachi TM -1000 at 10–15 keV acceleration voltage. No preparation for the samples was required for the image obtainment using this equipment.

### Statistical analysis

Analysis of variance (ANOVA) and Bonferroni *post hoc* test were carried out to detect the differences between the coating mass and antimicrobials delivery. P values < 0.05 were considered significant different. The software package SPSS (Version 20, IBM Corporation, Armonk New York, USA) was used for all statistical calculations. Prism 5 for Windows (GraphPad Software, Inc., La Jolla, CA, USA) was used to create the graphs.

## Results

### Pilot test for validation of coating method

**Figure 1:**
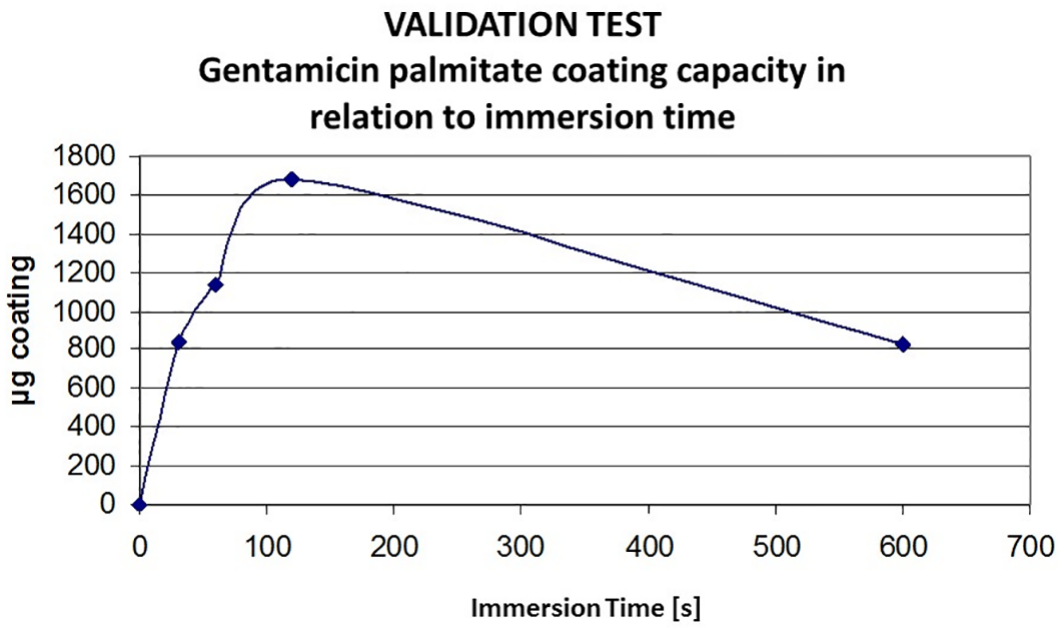
Validation of Gentamicinpalmitate coating capacity on Optilene in relation to immersion time. Optilene sample 1 x 1 cm; GP in MeOH; immersion in s, GP, gentamicinpalmitate.

Optilene^®^ showed higher coating capacity from 12% after an immersion period of 120s (**Figure 1**).

**Figure 2:**
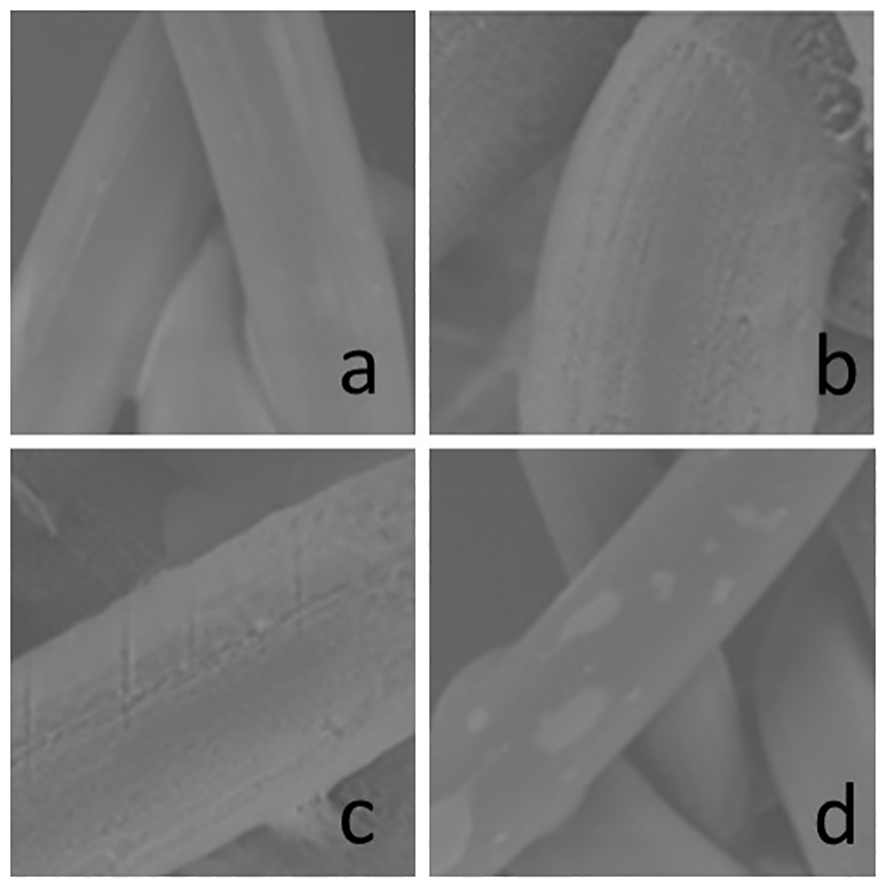
Scanning electron microscopy (SEM) of the Optilene^®^ meshes uncoated **(A, B)** and GP coated **(C, D)**. **(A)** uncoated x 600, 1,1cm = 100µm, **(B)** uncoated x 1500x, 1,1cm = 50µm, **(C)** coated x 1500, 1,1cm = 50µm, **(D)** coated x 600, 1,1cm = 100µm; scale bar is valid for **A-D**. GP, Gentamicinpalmitate.

Highest coating yield was obtained after 120 s immersion time, it was 1682 µg/sample. Meshes coated with GP showed an homogeneous distribution of the solution on the polypropylene. The GP coating showed an intense granulated surface over the biomaterial surface once the chlorhexidine coating was present in some isolated areas (**Figure 2**).

**Figure 3:**
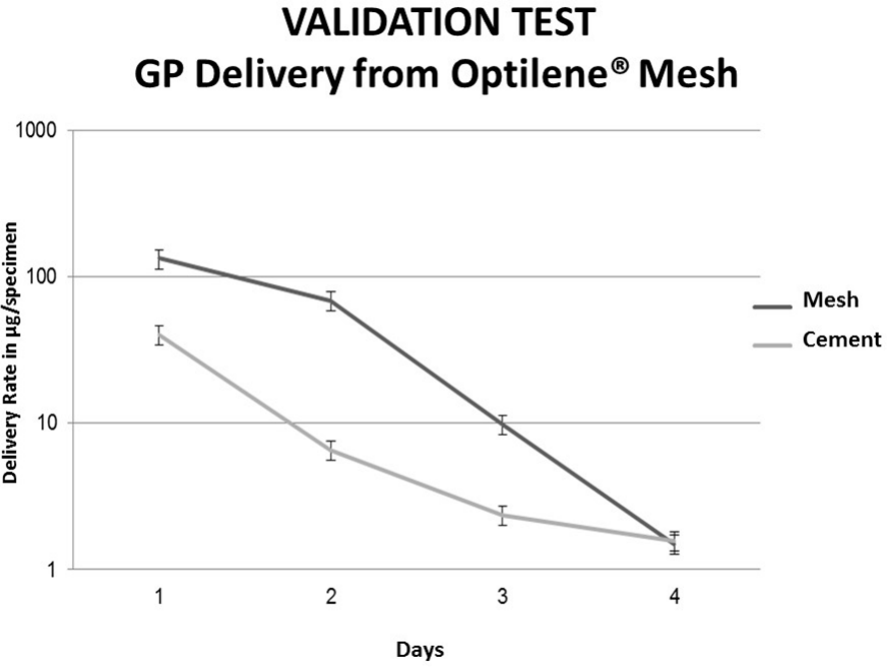
Comparison of cumulated amount of Gentamicin from GP coated Optilene and Palacos R+G; Palacos R+G (gentamicin containing acrylic cement); GP, gentamicinpalmitate coated Optilene sample = 1 x 1 cm; Palacos R+G Dynstat specimen (10x15x3mm); Palacos R+G in dark gray; Optilene + GP in light grey.

The delivery rate of GP from coated Optilene^®^ meshes reaches a high concentration during the first 24 h. The GP was not detected after 4 days. The release of Gentamicin was similar to that of Gentamicin from PMMA cement used, however the mesh surface presented almost no coating on its surface after four days (**Figure 3**).

**Figure 4:**
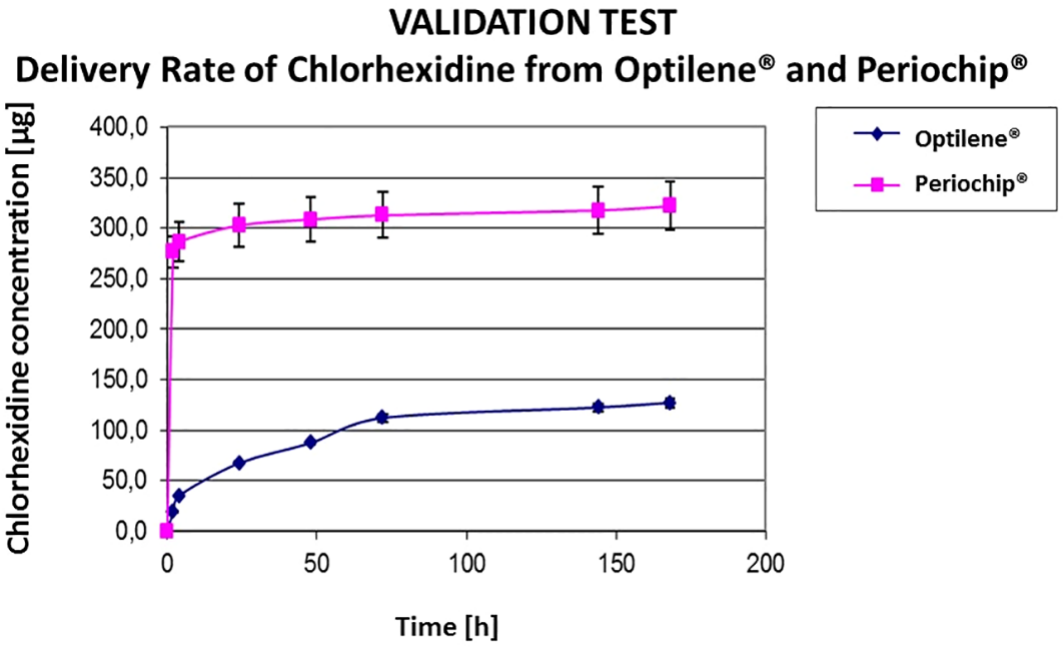
Comparison of Chlorhexidine release from Optilene and Periochip. Periochip contains 250 mg CHX-glyconate, Optilene contains; CHX, chlorhexidine; sample 1 × 1 cm.

Similar results were observed from Optilene^®^ meshes coated with chlorhexidine. We compared the chlorhexidine delivery rate from Optilene^®^ to chlorhexidine elution from Periochips^®^. The delivery from Periochip^®^ was significantly higher in the first minutes decreasing within the days. The delivery from Optilene^®^ meshes was not so high on the first minutes showing a continuous pathway until the end of 7^th^ day (**Figure 4**).

With this validation tests we can affirm that meshes coated with antiseptic allow the delivery over a longer period than meshes coated with antibiotics.

### Determination of the coating density of other hernia meshes

A dependency from immersion time in each coating solution was detected. Up to 200 s immersion time was investigated.

120 s immersion time were the optimum time for treatment with GP solution, for CHl-P and CHl-PA solutions the highest coating yields were observed after 200 s immersion time, independent from the underlying mesh material. For elution studies, the mesh samples were prepared with 200 s immersion time.

**Figure 5:**
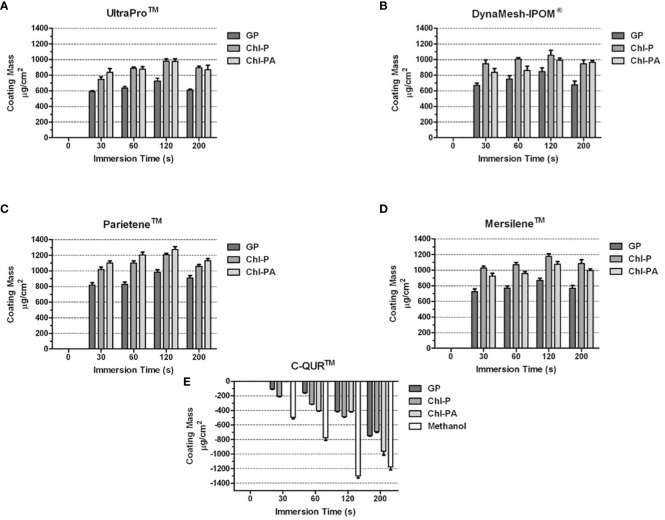
**(A–E)** Coating mass acquired by each mesh after immersion with GP, Chl-P and Chl-PA solution. **(A)** UltraProTM; **(B)** DynaMesh-IPOM^®^; **(C)** ParieteneTM; **(D)** MersileneTM; **(E)** C-QURTM.

Certain uniformity was observed on the quantity of chlorhexidine coating the surface of each mesh used. UltraPro™ and DynaMesh-IPOM^®^ were coated with 800–1000 µg/cm^2^ (**Figures 5A, B**) of Chl-P and Chl-PA while Mersilene™ and Parietene™ were coated with 1000–1200 µg/cm^2^ (**Figures 5C, D**) of Chl-P and Chl-PA approximately. The quantity of GP coating on the meshes was also higher on UltraPro™ and DynaMesh-IPOM^®^ (600–800 µg/cm^2^; **Figures 5A, B**) while on Mersilene™ and Parietene™ the amount of 800–1000 µg/cm^2^ (**Figures 5C, D**) was present. Due to original coating with bio-resorbable omega-3 the surface of the meshes contained high concentrations of fatty acids. Therefore, tests with the C-QUR™ meshes were not successful. Coating tests with C-QUR™ showed negative results. The amount of the omega-3 was dissolved from the surface, and the mesh lost its mechanical stability (**Figure 5E**).

### Antibiotic and antiseptic elution rate of other hernia meshes

**Figure 6:**
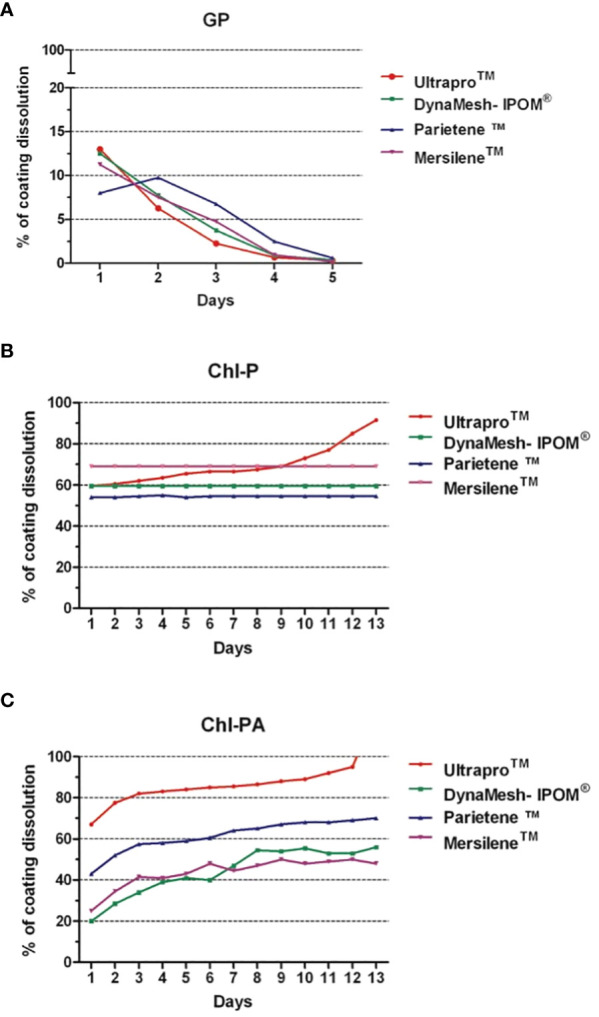
Percentage of coating dissolution. **(A)** GP; **(B)** Chl-P; **(C)** Chl-PA.

GP was detected until the 5^th^ day interval. The initial amount detected was around 100–150 µg/cm^2^ decreasing to 6–8 µg/cm^2^ at the day 5, only 10–15% of the GP used for coating the meshes. We did not detect significant difference between the amounts released from each mesh (**Figure 6A**). The release of Chl-P from all meshes was observed until 13 days after immersion. From DynaMesh-IPOM^®^, Parietene™ and Mersilene™ 55–65% of the total coating were released, while almost 100% was released from UltraPro™. The release of Chl-P from UltraPro™ and Mersilene™ were significantly higher (p<0.05) in comparison with the other two meshes (**Figure 6B**). Similar delivery was observed for Chl-PA. UltraPro™ released almost the total amount of coating until the 13^th^ day while the other meshes released between 50–60% of the total coat (p<0.05; **Figure 6C**).

### Scanning electron microscopy

**Figure 7:**
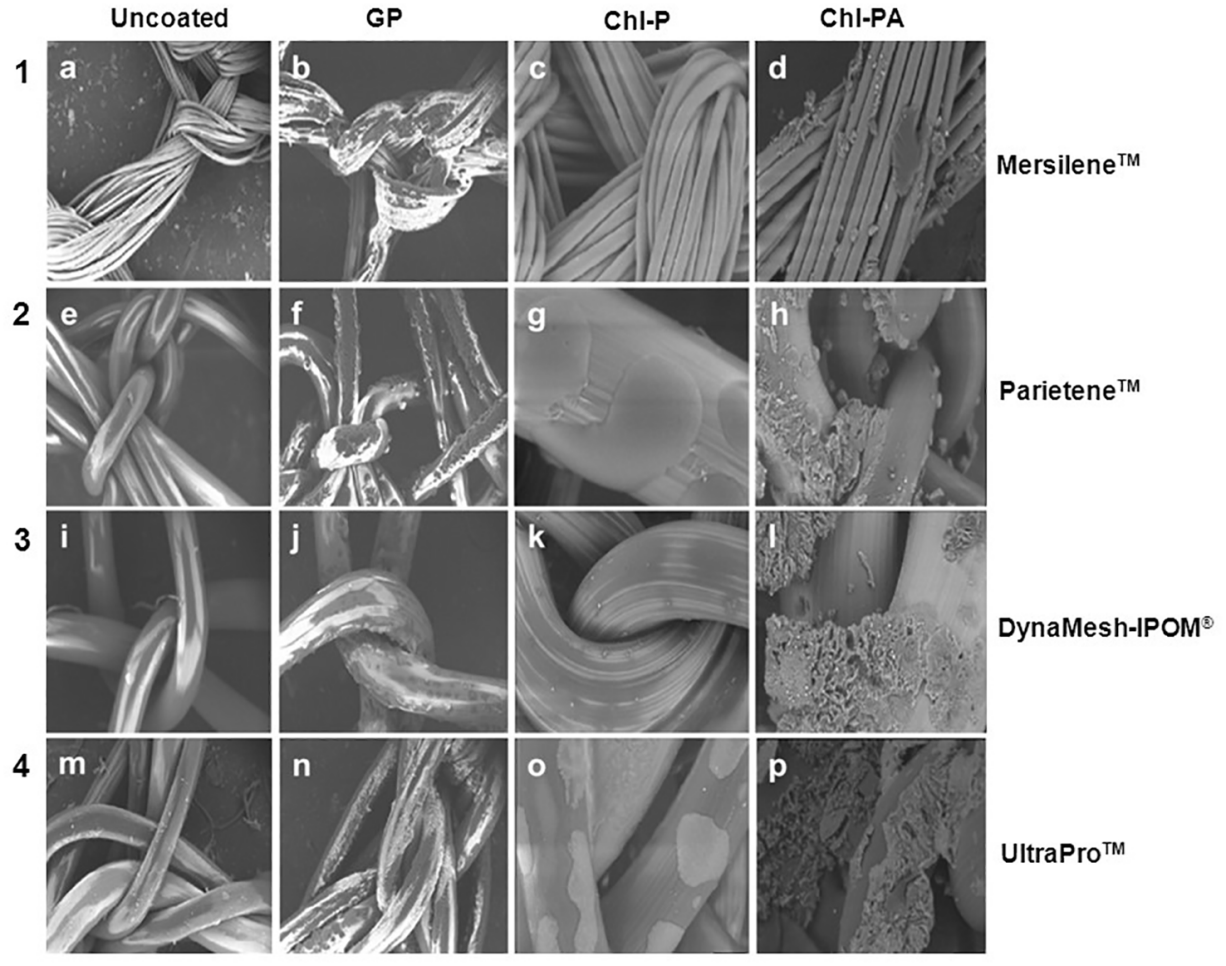
**(A–D)** SEM of polyester meshes (Mersilene^®^). **(A)** uncoated **(B)** coated with GP **(C)** coated with CHX-palmitate **(D)** coated with CHX acetate in palmitic acid. **(E–H)** SEM of polyester/polylactide meshes (Parietene^®^). **(E)** uncoated **(F)** coated with GP. **(G)** coated with CHX palmitate **(H)** Coated with CHX acetate in palmitic acid. **(I–L)** SEM of PVDF meshes (DynaMesh^®^). **(I)** uncoated **(J)** coated with GP **(K)** coated with CHX palmitate **(L)** Coated with CHX acetate in palmitic acid. **(M–P)** SEM of polypropylene-polyglecaprone meshes (UltraPro^®^). **(M)** uncoated **(N)** coated with GP **(O)** coated with CHX palmitate **(P)** Coated with CHX acetate in palmitic acid.

The SEM images show the surface modification between uncoated and coated samples (**Figures 7A, C, E, G, K, L, M, O**). The coating substances covered the surface of the fibers without damaging its structure. The coating substances were distributed all along the fibers in all samples. Higher uniformity could be observed on samples coated with GP in comparison with Chl-P and Chl-PA (**Figures 7B, F, J, N**).

Granules-like structures could be observed on samples coated with Chl-PA (**Figures 7D, H, I, P**). Due to the low mechanical stability on smooth surfaces, no images of C-QUR™ meshes could be observed by SEM.

## Discussion

However, mesh-related infections have been reported and they are of considerable clinical as well as of economic importance (**Table 1**). Deep surgical site infection (SSI) following mesh graft for hernia repair is a challenge for patients and surgeons. Antibiotic coating for indwelling medical devices has been carried out; however there are only few studies about coating hernia meshes with antimicrobial substances (Blatnik et al., 2017, Mirel et al., 2022).

**Table 1:** Economic overview of hernia surgery.

Costs/Use	Economic Potential
Number of surgeries in Germany	350.000
Infection rate	3%
Total cost per surgery	5750 – 6678 €
Additional costs to treat infections [Prolonged hospitalization: 7,3 days]	2308 €
Total costs per surgery with infection	8058 – 8986 €
Number of infection per year	10.500
Total economic potential per year	84.609.000 – 94.353.000 €

The coating was carried out by immersing different meshes in the antimicrobials solution and dried overnight.

With regard to GP coting solution, an optimum immersion time of 120 s was determined, a longer time period lead to decrease of coating load, probably caused by redissolution in methanolic coating solution.This method is suitable prior a surgical procedure taking into consideration that the same should be done in sterile conditions. This study showed that after 4 minutes of immersion the meshes were coated with an amount of approximately 1000 *µ*g/cm^2^ of each substance. We observed that the quantity of GP coating on the meshes was higher on UltraPro™ and DynaMesh-IPOM^®^ (600–800 µg/cm^2)^; than on Mersilene™ and Parietene™ (800–1000 µg/cm^2).^ The reason for this behaviour might be due to different material composition and surface structure as shown in SEM pictures. Due the hydrophobic profile of GP, Chl-P and Chl-PA the immersion allowed a rapid adherence of the antimicrobials on the biomaterials surface. Easy and rapid to be carried out, even with the drying time, this method would be a suitable for coating hernia meshes in prior hernia surgeries. Success on the use of GP, Chl-P and Chl-PA for coating biomaterials and bone samples for prevention and treatment of infections was already described by different authors (Matl et al., 2009; Coraca-Huber et al., 2013b).

The Mersilene™ meshes present finer filaments in its composition in comparison with the other meshes used in this study. That structure increases the surface area for the coating what can be observed on the SEM images as well as on the measurements of coating mass. The other meshes present a similar structure with thicker filaments (Najm et al., 2023). Also, the different coating substances interacted differently to the meshes. GP coats the filaments more homogeneity covering almost all surfaces in comparison with Chl-P and Chl-PA. Chl-P coat showed dots distributed along the fibers while Chl-PA looks like crumps and granules.

The amount of substances released from the meshes showed some variation according to each mesh brand and substance (Blatnik et al., 2017, Mirel et al., 2022). While all meshes showed a similar delivery of GP, UltraPro™ delivered more Chl-P and Chl-PA in comparison with DynaMesh-IPOM^®^, Parietene™ and Mersilene™. Also, Chl-P and Chl-PA showed a longer release term in comparison with GP. This finding shows that Chl-P and Chl-PA can be better coat substances for hernia meshes in contrast to GP. Chl-P and Chl-PA could be measured after 2 weeks of immersion (Majumder et al., 2015). Transferring it to the clinical scenario, a hernia mesh and surrounding tissue would be protected against infection for longer time comparing to GP coated samples.

Bactericidal tests as well as biocompatibility tests were not carried out in this study. However, studies with Chl-P and Chl-PA used as coating for suture samples showed superior bactericidal effect against *Staphylococcus aureus*. The same study testes the biocompatibility of suture coated by these substances with positive results (Matl et al., 2009). Taking it into consideration, we suggest the use of Chl-P and Chl-PA, as well as GP, for coating of hernia meshes aiming prevention of infections. Further investigation of the bactericidal effect of coated hernia meshes against biofilm form of *S. aureus* and other device-related infections is suggested.

The aim of the study was to combine a gentamicin (as palmitate) as well as chlorhexidine as a coating on the surface of hernia meshes. Both drug materials, gentamicin as well as chlorhexidine are well known in clinical practice. No biofilm formation occurred with gentamicinpalmitate coated implants tested according to ISO 17025 (proliferation assay). Furthermore, the gentamicin as palmitate is biocompatible according to ISO 10993 (Kühn and Brünke 2010). All coatings were very thin and incorporated in the rough surface of the meshes. We could not observe an influence of the coating on the stiffness and flexibility of the mesh. Immediately after implantation the meshes come into close contact with body liquids and the coating will dissolve (Mirel et al., 2022). Further investigations need to be carried out on the behavior an interaction of gentamicin and chlorhexidine with hernia mesh material under clinical application.

## Data Availability

The raw data supporting the conclusions of this article will be made available by the authors, without undue reservation.
